# A Virtual Counseling Application Using Artificial Intelligence for Communication Skills Training in Nursing Education: Development Study

**DOI:** 10.2196/14658

**Published:** 2019-10-29

**Authors:** Shefaly Shorey, Emily Ang, John Yap, Esperanza Debby Ng, Siew Tiang Lau, Chee Kong Chui

**Affiliations:** 1 Alice Lee Centre for Nursing Studies National University of Singapore Singapore Singapore; 2 Information Techonology National University of Singapore Singapore Singapore; 3 Department of Mechanical Engineering National University of Singapore Singapore Singapore

**Keywords:** artificial intelligence, communication, learning, nursing education, patients, technology, virtual reality

## Abstract

**Background:**

The ability of nursing undergraduates to communicate effectively with health care providers, patients, and their family members is crucial to their nursing professions as these can affect patient outcomes. However, the traditional use of didactic lectures for communication skills training is ineffective, and the use of standardized patients is not time- or cost-effective. Given the abilities of virtual patients (VPs) to simulate interactive and authentic clinical scenarios in secured environments with unlimited training attempts, a virtual counseling application is an ideal platform for nursing students to hone their communication skills before their clinical postings.

**Objective:**

The aim of this study was to develop and test the use of VPs to better prepare nursing undergraduates for communicating with real-life patients, their family members, and other health care professionals during their clinical postings.

**Methods:**

The stages of the creation of VPs included preparation, design, and development, followed by a testing phase before the official implementation. An initial voice chatbot was trained using a natural language processing engine, Google Cloud’s Dialogflow, and was later visualized into a three-dimensional (3D) avatar form using Unity 3D.

**Results:**

The VPs included four case scenarios that were congruent with the nursing undergraduates’ semesters’ learning objectives: (1) assessing the pain experienced by a pregnant woman, (2) taking the history of a depressed patient, (3) escalating a bleeding episode of a postoperative patient to a physician, and (4) showing empathy to a stressed-out fellow final-year nursing student. Challenges arose in terms of content development, technological limitations, and expectations management, which can be resolved by contingency planning, open communication, constant program updates, refinement, and training.

**Conclusions:**

The creation of VPs to assist in nursing students’ communication skills training may provide authentic learning environments that enhance students’ perceived self-efficacy and confidence in effective communication skills. However, given the infancy stage of this project, further refinement and constant enhancements are needed to train the VPs to simulate real-life conversations before the official implementation.

## Introduction

### Background

Effective communication skills are an integral part of the nursing profession and the foundation for high-quality nursing care [1]. Poor communication skills have been directly related to high turnover rates; low morale among nurses [2,3]; and poor patient outcomes such as medical errors, poor adherence to the treatment plans, and lower patient care satisfaction [4]. Effective communication between nurses and patients involving nurses’ abilities to explain, listen, and empathize is necessary for successful outcomes in individualized patient care [5,6]. However, nursing students are often stressed over their lack of adequate skills to communicate effectively with patients and their family members [7,8]. This indicates a deficit in the availability of specialized communication training for nurses [7-9] and the ineffectiveness of the current communication skills training for nursing undergraduates through didactic lectures [10,11].

The need for communication skills training to be both participatory and experiential [11] led traditional nursing curricula to use simulated or standardized patients as tools to help students to develop clinical reasoning, patient communication, history taking, physical examination, and patient diagnosis skills [12,13]. Standardized patients are community members who are carefully recruited and trained to take on the characteristics of a real patient, and they provide students with opportunities of learning and assessments in simulated clinical environments [13]. However, the development and maintenance of a quality standardized patient program is costly and time-consuming [14]. Moreover, standardized patients are often subjected to feelings of anxiety, fatigue, physical discomfort, and biasness, which carry some reliability concerns [15,16]. Therefore, virtual patients (VPs) might be a more viable alternative.

VPs are computer-based simulations of authentic clinical cases that allow users to interact with the system for the purpose of health care or medical trainings, education, or assessments [17,18]. Current uses of VPs in medical education are primarily to develop students’ clinical reasonings, problem-solving skills, core or conceptual knowledge acquisitions, skills acquisitions, and affective characteristic developments (eg, professional competence) [19]. Reviews evaluating the effectiveness of VPs in medical education [17,20] have reported VPs as a cost-effective tool and as successful in facilitating clinical reasonings, communication skills, and ethical reasonings among students when used as an alternative or supplementary tool to existing curricula. However, in studies in which VPs have been used to teach and assess interview skills, students recognized the artificiality of such situations and did not demonstrate empathy or other important aspects of this skill [21,22]. Despite a few flaws in comparison with standardized patients, the use of VPs in medical education provided secured learning environments and opportunities for extensive repetitive practice with feedback and without negative consequences to real or standardized patients [21,23]. Apart from the acquisition of clinical knowledge and skills, VPs also provide students with opportunities for self-directed learning [24], which leads to reflection [25] and self-driven change [26,27]. Given their advantages and effectiveness for student learning outcomes, VPs are therefore more extensively used in medical education, although their use in nursing education is still limited [28].

### Theoretical Framework

The theoretical frameworks that guided this study are Bandura’s self-efficacy theory [29] and Herrington et al’s [30] authentic learning concept. According to Bandura [29], self-efficacy is an individual’s confidence about one’s ability to carry out a behavior effectively, and this influences one’s motivation and efforts in performing a specific task, individual goal setting, and perspective [31]. Therefore, self-efficacy is an essential component needed by nursing students to display effective communication skills in health care settings. Moreover, if students have higher perceived self-efficacy in their communication with patients, their family members, or other health care providers, they may be more inclined to initiate and engage in conversations, which can boost patient-provider and work relationships.

**Figure 1:**
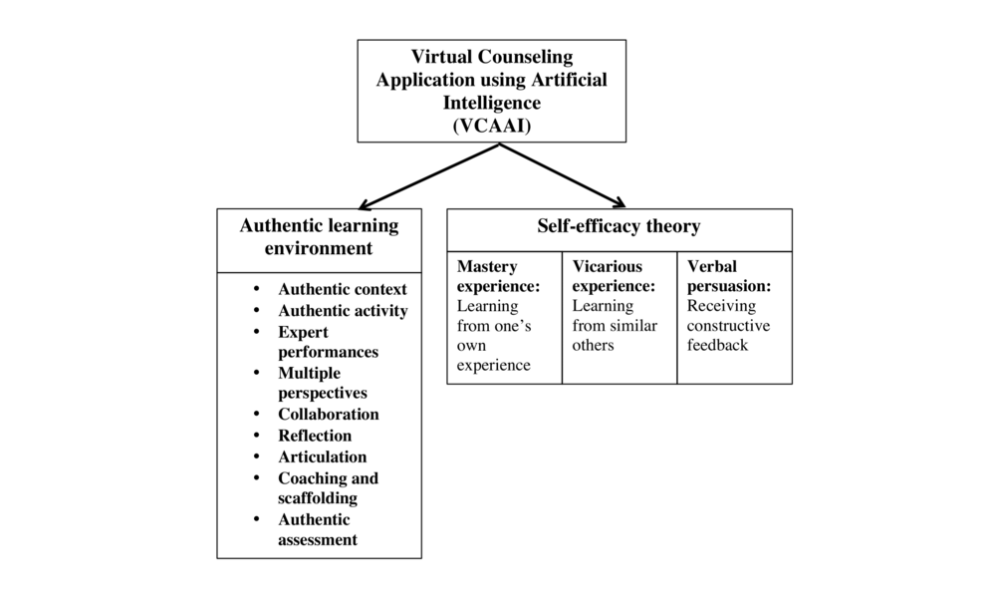
Theoretical framework for virtual counseling application using artificial intelligence.

Bandura also mentioned the importance of mastery experience (learning from one’s own experience) and verbal persuasion (receiving feedback on one’s own performance) in enhancing an individual’s self-efficacy [29]; therefore, these factors were taken into consideration when planning the VP case scenarios. In addition, 9 elements of authentic learning environments [30] consisting of authentic contexts, authentic activities, expert performances, multiple perspectives, collaboration, reflection, articulation, coaching and scaffolding, and authentic assessments will be introduced during the program planning to provide authentic exposure and encourage real-life learning to prepare nursing students for their future professional lives. The theoretical framework of this study is presented in Figure 1.

### Preliminary Study

This project is an extension of a preliminary study conducted in 2016 in which a blended learning approach was adapted to teach communication skills to year 1 nursing undergraduates [32,33]. The redesigned course retained face-to-face tutorials and replaced didactic face-to-face lectures with electronic lectures. Web-based quizzes, discussion forums, and reflection exercises were introduced to enhance students’ engagement with the Web-based course material. To promote applications of the theoretical contents, face-to-face tutorials included role-play and problem-based learning using authentic clinical scenarios (ie, standardized patients) to simulate conversations between nurses, other health care professionals, patients, and their family members. Findings suggested that students who received the module via the blended learning approach had improved communication self-efficacy, better attitudes in learning communication skills, statistically significantly higher satisfaction, and better academic scores compared with participants from the previous cohort who had only didactic lectures [32]. Apart from enhanced learning, students also reported confidence boosts in handling similar situations in their year-end assessments, but they were less confident in retaining these learned skills and transferring it to real-life clinical settings during their end-of-semester clinical postings [33]. Stakeholders also mentioned deterioration in communication skills among year 3 and 4 nursing students, which indicates a necessity for additional resources to reinforce their communication skills. However, employing standardized patients, as such for year 1 students, to provide authentic communication training in subsequent years is expensive and resource extensive. Therefore, these findings motivated the development of VPs to fill in the previous gaps and provide nursing students with continuous authentic trainings in communication skills. Furthermore, the use of VPs also aligns with the university’s goal of encouraging self-directed and autonomous learning among students using electronic platforms.

## Methods

### Overview

The Alice Lee Centre for Nursing Studies under the National University of Singapore (NUS) offers a 3-year (4 years for honors students), full-time Bachelor of Science (Nursing) program that is accredited by the Singapore Nursing Board. The course covers core modules, such as anatomy, physiology and physical assessment, pathophysiology, pharmacology and nursing practice, communication, and cultural diversity, and includes clinical practicums at tertiary hospitals that range from 2 weeks to 3 months. This project will be conducted with nursing undergraduates of the NUS who have completed the core module *Effective Communication for Health Professionals* (module code NUR1110) in the year 1 of their nursing courses. The 2-year study will follow these students in year 2 and year 3 consecutively by introducing VPs depicting real-life case scenarios at gradual difficulty levels before their end-of-semester clinical postings. A total of 4 VP case scenarios were developed for each semester on the following topics: (1) interviewing a pregnant woman with pain to solicit holistic history taking (year 2 semester 1); (2) history taking from a depressed patient (year 2 semester 2); (3) using a standardized approach such as situation, background, assessment, and recommendation (SBAR) to hand-off interdisciplinary communications (year 3 semester 1); and (4) showing empathy to a fellow nursing student (year 3 semester 2). Overall, the aim of this project was to develop and evaluate the use of VPs to better prepare nursing undergraduates in communicating with real-life patients, family members, and other health care professionals during their clinical postings. The specific research questions we plan to answer in this project are as follows:

1. What is the effect of using VPs in enhancing nursing undergraduates’ self-efficacy and attitudes toward learning communication skills?
2. Do the students who receive additional training using VPs perform better in their communication skills during their clinical postings compared with students who receive standard training?
3. What are the levels of outcomes of students’ self-efficacy and attitudes toward learning communication skills at pretest (year 2 semester 1 before receiving VP training), posttest 1 (last day of clinical posting year 2 semester 1), posttest 2 (last day of clinical posting year 2 semester 2), posttest 3 (last day of clinical posting year 3 semester 1), and posttest 4 (last day of clinical posting year 3 semester 2)?
4. What are the changes in self-efficacy and attitudes toward communication skills scores over time (pretest and posttests 1 to 4)?
5. What are the students’ experiences in receiving additional training using VPs before their clinical postings?

The aim of this paper was, therefore, to provide a detailed breakdown on the development process of the virtual counseling application using artificial intelligence (VCAAI) for communication skills training in nursing education and to highlight recommended resolutions and challenges faced to inform future research.

### Design and Development

The research team involved in the design and development of the VCAAI comprises clinical nurses, nurse educators, and information technology (IT) experts. On the basis of the vast experience of the research team, the detailed dialogue flows with mind maps were written at the initial stage. The Master Interview Rating Scale (MIRS) provided a framework to develop the scenarios. The MIRS was designed to teach effective communication between health care practitioners and patients. It has been used in medical education over the past decades [34]. On the basis of the 15 basic items in the MIRS framework, the team created a coding schema to classify the characteristics of a patient’s interview questions into 1 of the following categories: open-ended, close-ended, empathetic statements, information gathering, and the patient’s perspective.

The nursing research team then worked closely with the technology team from the NUS Information Technology department to develop a voice chatbot learning system. The voice chatbot was powered by an artificial intelligence (AI) using the natural language processing engine Google Cloud’s Dialogflow. The AI uses artificial neural networks just like human intelligence to learn from varying situations to recognize, classify, and predict responses based on analysis by machines such as computer systems [35]. In this study, a limited memory AI [36] was used to mimic the human characteristics of standardized patients used in simulation training for communication skills. The standardized patient’s conversations were further visualized in a three-dimensional (3D) avatar form, characterized by natural, nonverbal gestures to elicit more engaging connections as well as to have more life-like realism. It was later further integrated and visualized into a 3D avatar form to mimic human conversations (both verbal and nonverbal) by leveraging Unity 3D, which is a popular 3D development platform. According to Hintze et al [36], a limited memory AI uses past experiences to inform future decisions. As such, our 3D avatar (VP) was trained using Google Cloud’s Dialogflow processing engine to store memories of potential conversations. Supervised learning by the machines (VPs) was modeled directly based on the research team’s observations and an analysis of live standardized patient interactions (video recordings) with the nursing undergraduates from our previous preliminary study.

### Case Scenarios

Students can interact with the VPs through 4 case scenarios, which were developed by members of the clinical nursing team with input from nursing students with a clinical background. These scenarios are based on authentic clinical cases (adapted from real-life clinical case studies), focusing mainly on communication aspects. The topic details and student objectives for the 4 scenarios described in Textbox 1.

The four scenarios were developed based on the growing clinical needs of nursing undergraduates. Each scenario is matched with the core modules students take during their undergraduate years. For example, the *pregnant woman* scenario is matched with the core module *Women and Child Health* that students take in year 2 semester 1. The *depressed patient* scenario is matched with the *Mental Health* module that students take in year 2 semester 2. The *SBAR* scenario is matched with the year 3 semester 1 module *Operating Theatre Nursing*, and the last *empathy* scenario is matched with the final year 3 semester 2’s clinical postings before students transit to clinical settings and registration with the Singapore Nursing Board.

**Case scenarios.**

Scenario 1: A 32-year-old pregnant woman in her third trimester experienced pain along the side of her belly while heading to the market in the morning. She was later diagnosed with Braxton Hicks because of a lack of consistent contractions and no increase in contraction strength. In addition, she is prone to menstrual cramps and has a history of miscarriage, which has rendered her very anxious, scared, and worried about her pregnancy. She does not have any children currently, and her spouse is often very busy.

- Objective: Students are required to interview the patient and find out more about her pain. Using the pneumonic *c*haracteristics of the pain, *o*nset of the pain, *l*ocation of the pain, *d*uration of the pain, *s*everity of the pain, *p*recipitating factors that make the pain worse or make her feel better, and *a*ssociated symptoms (COLDSPA), students will communicate with the pregnant woman to obtain a holistic idea about her pain and mutually develop her care plan to allay her anxiety.

Scenario 2: A 34-year-old, single, Chinese male lorry driver self-admitted himself to the hospital because of a relapse of depressive symptoms. He is in a depressed mood and has a closed-off demeanor, yet is fidgety and actively avoids eye contact. He has a history of major depressive disorder and was previously admitted because of a failed suicide attempt. He has undergone cognitive behavioral therapy and has been prescribed fluoxetine. Although his condition was stable at discharge, he is noncompliant with his prescribed regimen of fluoxetine and now complains of a loss of appetite, underproductivity, avolition, and reclusiveness.

- Objective: Students are required to establish a working rapport with the patient and elicit background information from a biological, psychological, and sociological perspective using the MIRS and relevant components of COLDSPA. Students will then proceed to develop a care plan based on the information they gather.

Scenario 3: A 42-year-old male patient was admitted to the general surgical ward after an operation (post appendectomy) 3 days ago. The student is assigned to change the patient’s operative wound dressing, but the dressing is soaked in blood, and he appears to be slightly pale, lethargic, and distressed.

- Objective: Students need to use the pneumonic SBAR to update the patient’s condition to the physician as well as to demonstrate clinical reasoning and appropriately prioritize areas of concern in patient care.

Scenario 4: The student user will take the role as a final-year nursing student communicating with another student (VP) during her final preregistration clinical posting period. The student will be very stressed because of escalating demands and clinical workloads.

- Objective: The student user will speak to this stressed student to find out more about her stressors and help her to reflect on how she can cope better. This scenario focuses on the principles of *Showing Empathy* using the pneumonic *N*aming emotion, express *U*nderstanding, showing *R*ecognition, and offering *S*upport (NURS) and on helping someone obtain his or her own perspective using the pneumonic *I*deas or beliefs of cause of the situation, how the situation is affecting daily *F*unction, and what *E*xpectations one has from the interviewer over the situation (IFE). These pneumonics have been taught in the *Effective Communication Among Health Professionals* core module, and the students should be well-versed in their use.

### User Interface

**Figure 2:**
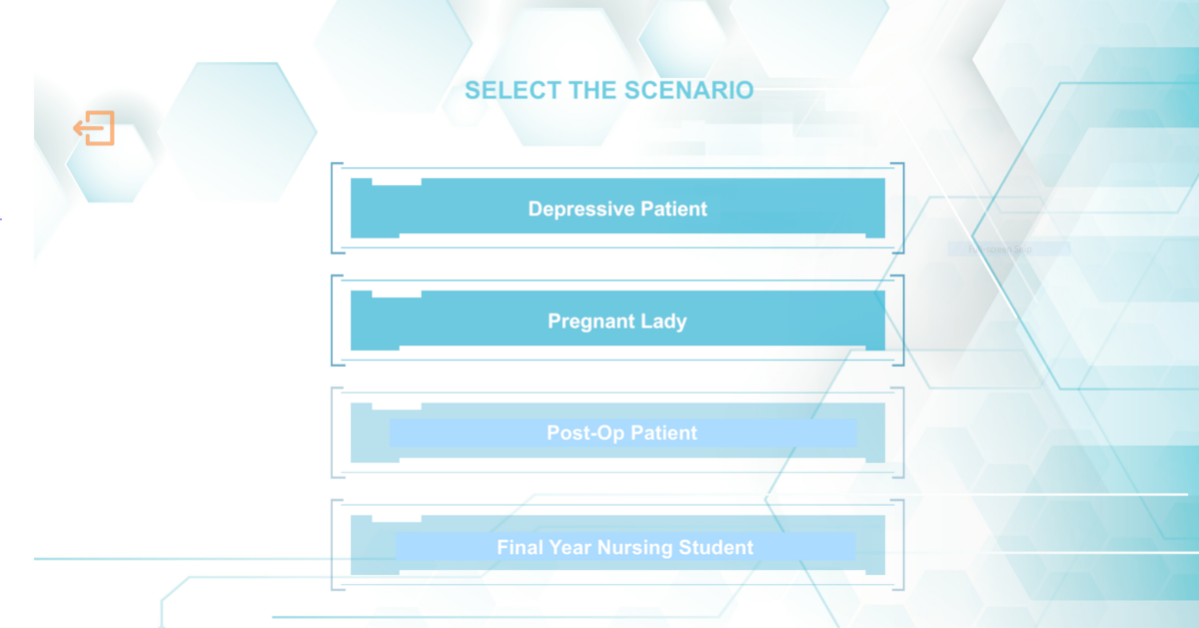
Case scenario selection menu for the virtual counseling application using artificial intelligence.

The VCAAI user interface was designed to be straightforward and user friendly with realistic features and detailed instructions to guide the students and optimize their user experiences. At the start of every session, students will arrive at the case scenario selection menu to choose 1 of the 4 scenarios mentioned previously (Figure 2).

**Figure 3:**
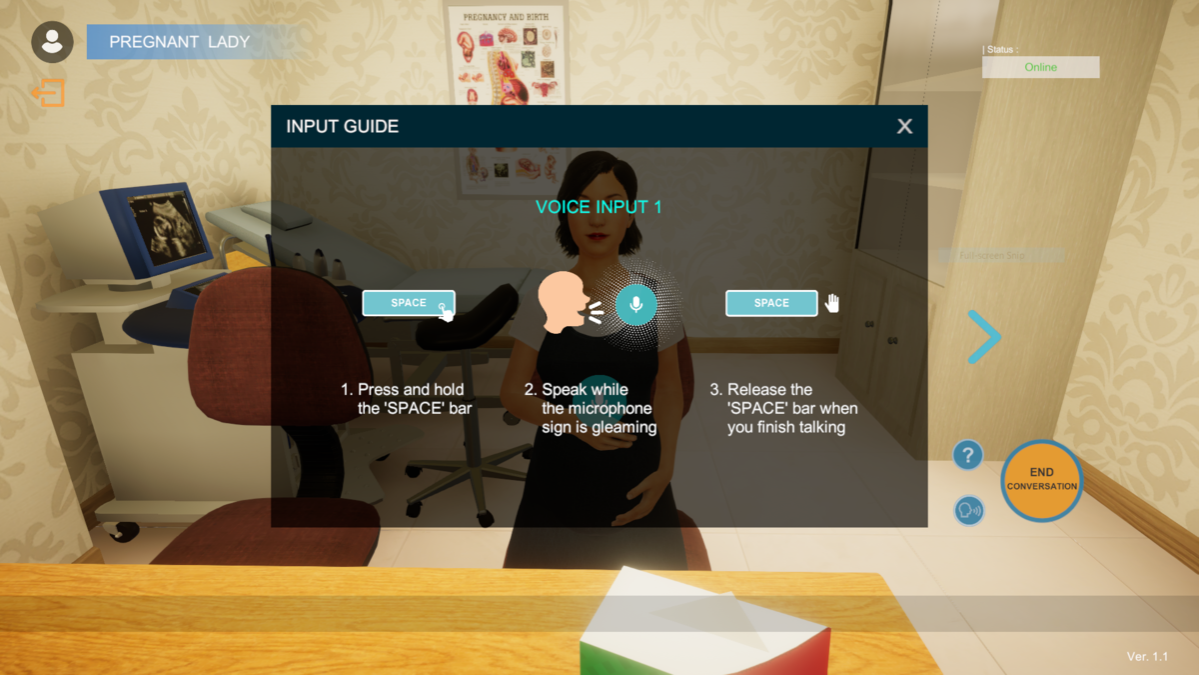
Technical voice input instructions on how to initiate a conversation with the virtual patient.

**Figure 4:**
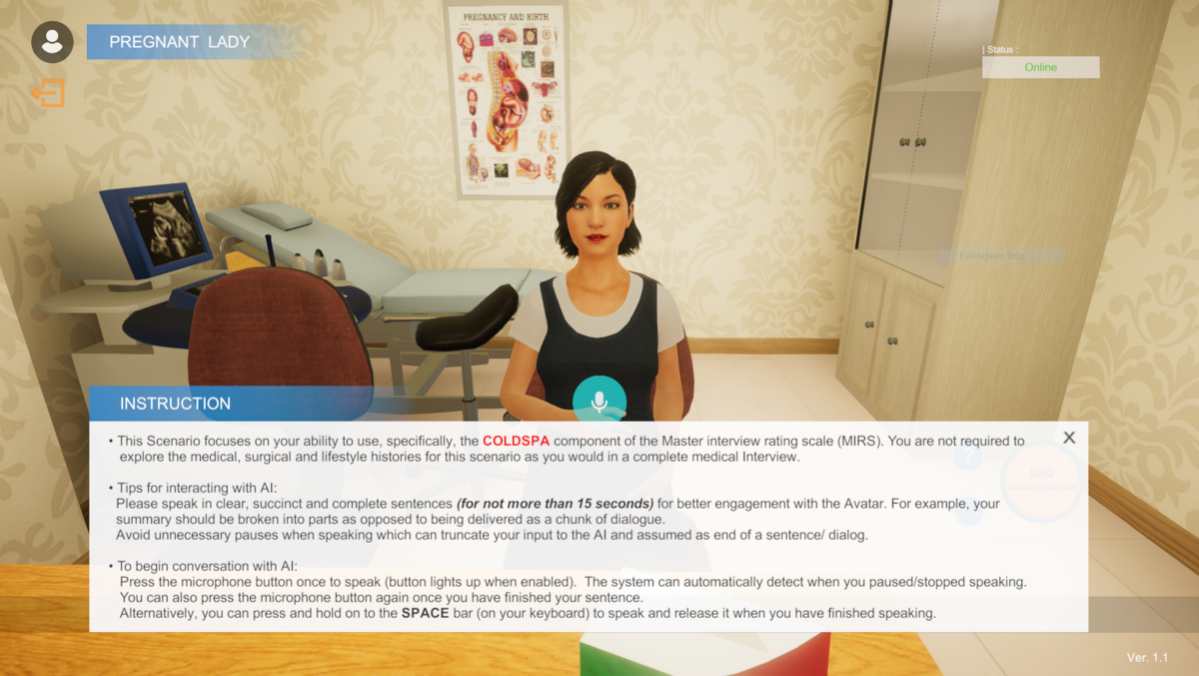
Assessment instructions and objectives for students.

The cases of the pregnant woman and the depressed patient were selected for illustrative purposes. Students will be first provided with introductory instructions to begin conversing with the completed 3D voice chatbot (Figure 3), after which they will be given interview objectives and instructions depicting their roles and relationships to the patient, which are guided closely by the context of the case (Figure 4).

**Figure 5:**
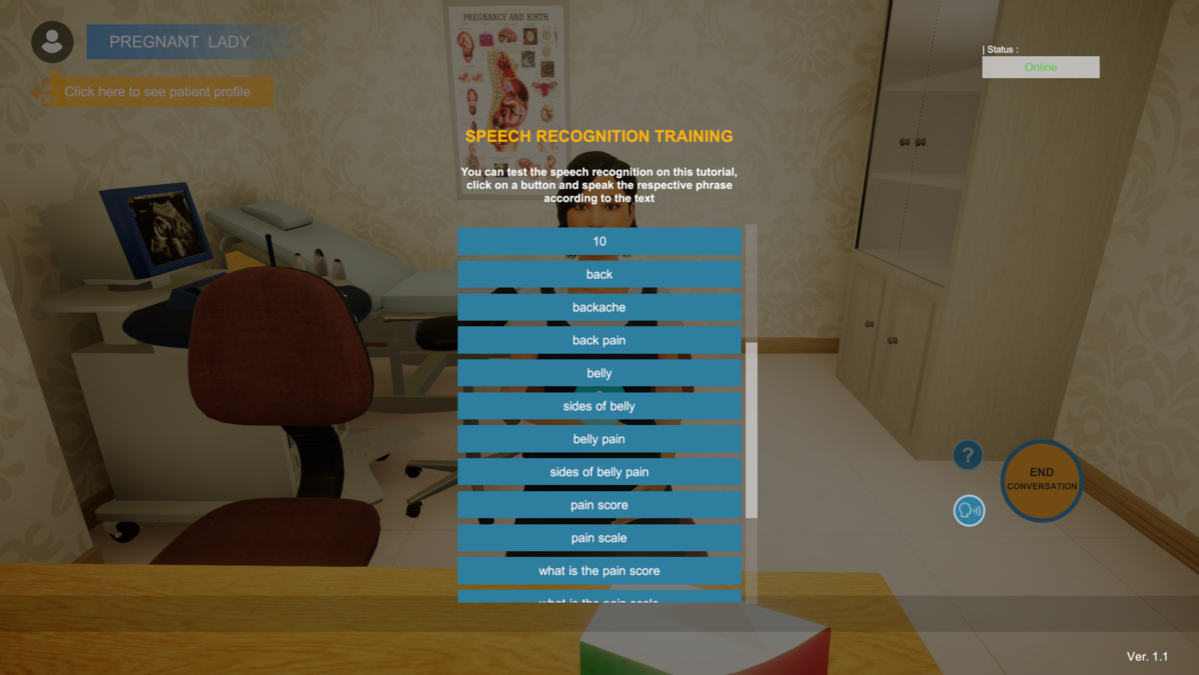
List of case scenario–specific keywords provided for voice recognition training.

Before interaction with the VP, students must complete a speech recognition training involving the main keywords related to the selected case scenario (Figure 5).

**Figure 6:**
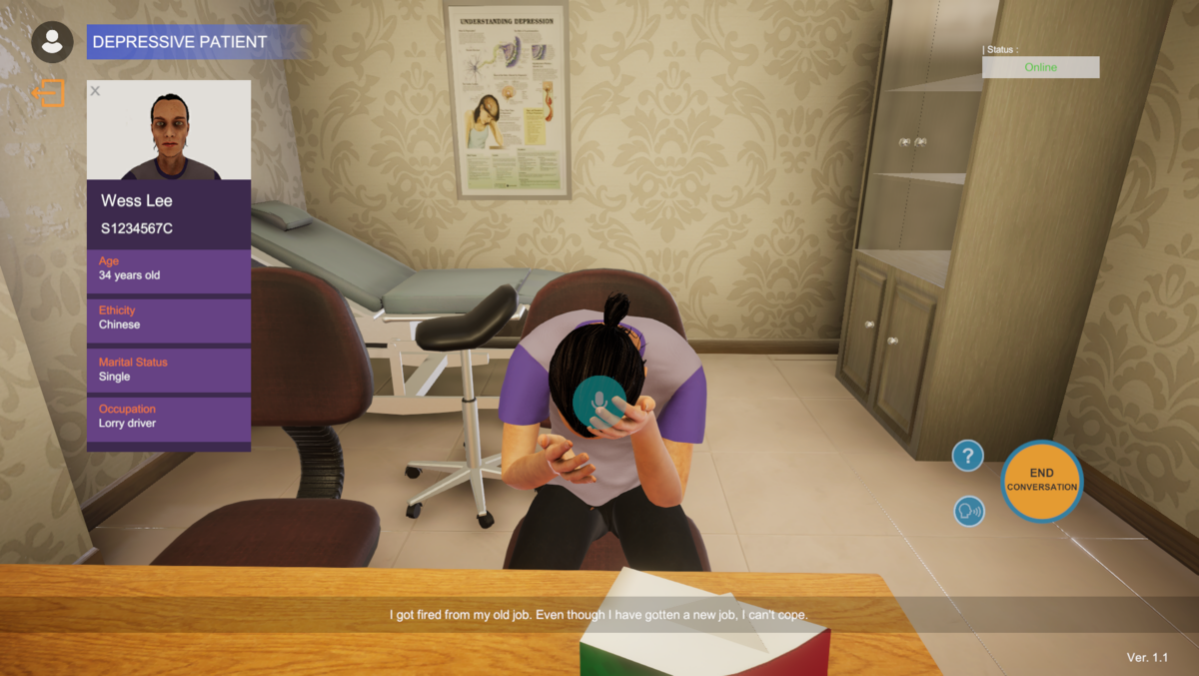
Case scenario of a depressed patient.

Upon successful pronunciations of a few keywords, students can proceed to interview the VP as per the nursing conversation guidelines (MIRS, COLDSPA, SBAR, NURS, and IFE). They will be presented with a first-person perspective of the patient avatar in 3D with his or her demographic profile and a list of potential questions to interact with the VP (Figure 6).

**Figure 7:**
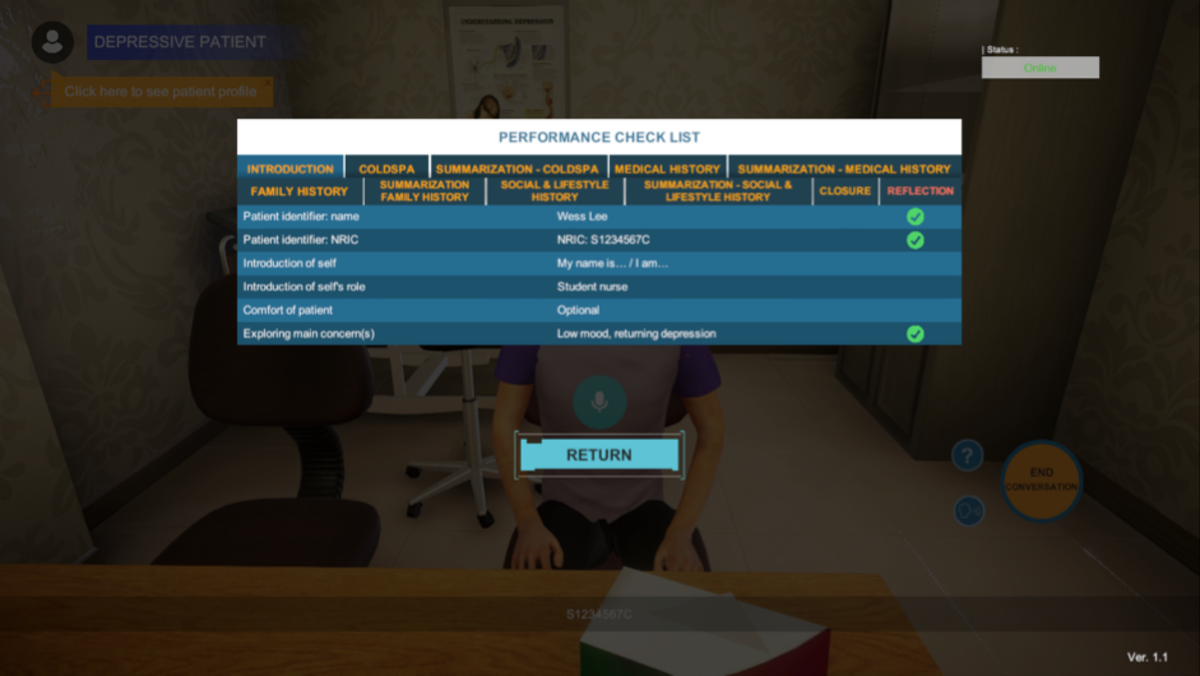
Performance checklist at the end of the assessment.

The list of questions is compiled from reviewing previously video-recorded interactions between standardized patients and nursing undergraduates while they interview these patients. These interviews are part of the year 1 nursing undergraduate core module titled *Effective Communication Among Health care Professionals’ Final Assessment*. The users have complete freedom to determine the sequence of the interviews. Nonverbal cues, such as the head-in-the-hands gesture, and a realistic clinical environment with relevant posters and equipment are incorporated to enhance the simulation of a real-life scenario. The scenario will end with a case summary discussing the main points shared, a performance checklist that serves as a formative feedback on their communication skills and interview processes, and a closure to recommend the next series of actions to take (Figure 7).

## Results

To test and validate the content, the VCAAI will be implemented on approximately 150 year 2 and year 3 nursing undergraduates. A user acceptance test (UAT) will be performed to evaluate the program’s effectiveness on nursing students’ performances, attitudes, and perceived self-efficacy pertaining to communication skills. Only nursing undergraduates who have completed the redesigned NUR1110 (Effective Communication for Health Professionals) core module will receive additional training via VPs in each semester (2 semesters per year) of year 2 and year 3 before they go for their clinical postings. Students will have unlimited access to the VPs (available scenarios depend on which semester the student is in) by logging in through the school portal. At the end of the semester, students’ performances, attitudes, and self-perceived self-efficacy in terms of communication skills will be measured using self-administered questionnaires, and the consolidation of students’ experiences and feedback will be done through focus group discussions. Approximately 20 clinical instructors (the exact number will be based on data saturation) supervising the nursing students during their end-of-semester clinical postings will also be interviewed for their perspectives of the students’ communication skills abilities. Subsequently, VPs users’ comments and ambiguities generated from the focus group discussions will be incorporated into the VCAAI program to further refine and optimize the user experience. Only then can the VPs be officially implemented and made available to all nursing students on the academic portal.

## Discussion

Throughout the course of development to the execution of the scenarios of the VCAAI program, the team met with various technological limitations and challenges to prove the effectiveness of the VPs, to imitate a standardized patient, and to provide smooth role-playing experiences for students. The context of these challenges and resolutions are categorized as follows: (1) content development, (2) technological limitations, (3) expectation management and contingency planning.

### Content Development

Content development entails permutation development, presentation of permutation, the relay of permutation to program designers for development, and the ability to predict possible dialogue permutations. Each of these aspects is important to ensure that the user experience with the VPs is fulfilling and beneficial.

A prominent issue that arose at the initial stage was that programmers and content developers spoke in different *languages* because of a lack of expertise in each other’s field. Content developers were focused on nursing communication skills, but programmers were unfamiliar with nursing concepts, conversation guidelines (MIRS, COLDSPA, SBAR, NURS, and IFE), and what a conversation between a nurse and a patient would be like. In addition, content developers did not know what information the programmers required and were unfamiliar with the technical terms used by programmers, and hence, they had difficulty translating their knowledge into *programmers’ language*. This difficulty in communication put restraints on the instructions and expectations of the VPs. Both teams should be more mindful when using technical jargon so that everyone can be on the same page, and technicalities should clearly be spelled out so that misunderstandings can be prevented. For example, the content developers can provide an information sheet that clearly explains to the IT team and vendor the common terms and expectations of health care communications in various scenarios. Visual diagrams and specific examples (eg, mind maps, drawings, and sample dialogues) were also useful in helping the programmers understand the content developers’ expectations of conversations. Such information sheets should be provided by both teams, which will give each other greater insights of each field, and this document should be kept accessible for easy reference when working individually. In addition, for the content developers to provide adequate information for the program developers, the programmers must create specific templates and constantly scrutinize the data before the data can be translated into the VPs. Moreover, administrative details such as the labeling of the files and the color coding of the data had to be mutually agreeable and adhered to by both parties.

To bridge the gap of expertise and information between content designers and IT experts, it will help to have a team member who encompasses expertise in both content design and IT. Frequent check-backs of the program process is needed through weekly face-to-face meetings. The communication styles of the nursing team (content developers) and the IT experts differ, and the keywords used to describe similar concepts vary, leaving room for misunderstandings and confusion, which can eventually cause distrust and frustration toward other team members. To resolve this issue, content developers and programmers maintained open communication and corresponded closely through mutual platforms (ie, emails and WhatsApp). Weekly face-to-face meetings were also conducted to prevent miscommunication through emails and WhatsApp messages. However, with the development of subsequent scenarios, there were marked improvements in terms of communication between team members and ease in the development process, which could be attributed to familiarity with the project by both teams.

To further improve the workflow between content developers and IT experts, a direct discussion with the actual program developers may be more effective than discussion with a representative. In this project, with special circumstances, the program developers were in a different country with a different time zone from the content developers, and the weekly meetings were communicated via a representative of the IT expert team who used an online chatting platform to relay the team’s message to the developers. This left room for miscommunication. Hence, it would be ideal for the program developers to be present more frequently during meetings and test runs so that they can experience the realities of the way the team communicates as well as bot performances with a user. It will also paint a clearer picture for them when they make amendments. However, given the tight budget, it was not feasible to fly them in on a weekly or even biweekly basis. The time zone differences also made organizing Skype calls difficult. For future collaborations, the availability and presence of on-the-ground developers can be considered before engaging an IT expert team.

Another challenge faced by the team was their inability to predict the possible intent of the conversations. Although content developers were focused on the components of nursing conversation guidelines (MIRS, COLDSPA, SBAR, NURS, and IFE) and various possible imputations, they failed to consider other conversational intents that went beyond the guidelines, such as small talk and giving advice. As students go beyond the scope of the guidelines, the content developers have major difficulties keeping up with the infinite possibility of inputs. This renders the conversations with the VPs disruptive and thus fails to effectively provide an immersive experience of role-playing for students. A resolution of this issue involved including senior nursing students, who had previously taken this communication module, in the VP testing to provide content developers with more permutations to broaden the VPs’ abilities to have natural conversations. However, the team was still met with the issue of keeping up with unexpected inputs by students, as seen in the UATs. Therefore, to encode the VPs to reflect the complexity and multifaceted nature of a person, programmers will have to constantly update the VPs using past inputs provided by the users. This process needs to be ongoing for at least one school semester, given the complexity of a natural conversation. Despite all the enhancements, there will always be some limitations for the VPs to account for because of countless unpredictable permutations. A continuous training of the VP will be needed to exhaust the maximum amount of data permutations with as many UATs as possible to report visible improvements only after each iteration of such a training in a longitudinal study. As such, VPs may be more effective with structured conversations and predictable inputs. For example, in the third scenario, as SBAR is more structural than conversational compared with the rest of the scenarios, the content design was relatively more linear and straightforward. Furthermore, playing as a fellow health care colleague receiving information from the user, the bot’s responses were often acknowledgment statements such as *okay* and *alright*, making content design a lot simpler. Upon testing within the team members, the conversation flow was indeed a lot more predictable and compartmentalized, and the majority of the responses from the program were appropriate.

### Technological Limitations

The VP is unable to adapt to the context of the conversation as the basis of communication for the VP is mostly reactive to the input, and it retrieves only a specific response tagged to the permutation. For example, in the first scenario:

Student: On a scale of zero to ten, can you rate your pain [belly pain]?

VP: Seven out of ten.

Student: Do you have any other pain?

VP: My back aches a little too.

Student: On a scale of zero to ten, can you rate your pain [back pain]?

VP: Seven out of ten [although the intended pain score for back pain was two out of ten to trigger prioritizing issues].

As seen, the keyword *pain score* was tagged with the answer *seven out of ten* as the belly pain was the main chief complaint. The VP was unable to adapt to the context, and this resulted in difficulties for content developers in tagging keywords to responses as similar questions can refer to different issues that require different responses.

Another example is the fourth scenario, which explores the use of IFE and NURS. As the program recognizes keywords to determine the appropriate responses, users may say *you seem stressed* with the intention of naming the emotion, and the keywords here are *seem* and *stressed*. Users may also say *don’t be stressed* with the intention of consoling, and the keywords are *don’t be* and *stressed*. With very similar keywords, the program may sometimes respond inappropriately, switching up responses for these 2 variations. With limited knowledge on how the program recognizes keywords or associates keywords together, content creators sometimes choose keywords inappropriately, resulting in the program being unable to fulfill its function. Such loopholes are easily missed out unless variations of the keywords are tested out with the program, the transcripts of the test runs are reviewed, and the content is amended afterward. This is manpower-intensive and time-consuming and may not be the most effective way to move forward. The possibility of the program learning variations and logic can be explored through predictions of speech and language.

Another key concern was that not all computers (eg, Mac, older versions of Windows, and Windows without Cortana or a graphic card) are compatible with the application, and certain earpieces or microphones do not work with the application. This led to a poor recognition of voice inputs and testers’ dictions, especially when a diction is heavily influenced by culture and content and the software used to develop the VPs uses American English. The software’s inability to detect, recognize, and translate speech to text effectively poses a challenge. There are various possible factors such as background noises and poor microphone qualities, but through the team’s observation, the students’ dictions were the primary issue. As Cortana (Microsoft Corporation), a voice recognition software, uses either American English or British English, if the students are more comfortable with Singlish (English-based creole or patois spoken colloquially in Singapore), the program will have difficulty recognizing their pronunciations or speech patterns, resulting in translation failures. For example, in addition to unique and complex medical terms (eg, laparoscopic appendectomy and postoperation day), when students attempt to mimic the Singapore setting (ie, by naming various patients with common Singaporean names), the translations can be inaccurate. Moving forward, to counter text translation difficulties, audio-recordings of real Singaporean users will be incorporated into the program to enhance text translation abilities and to eliminate some errors from varying accents and pronunciation differences between Singaporean English and American English. In addition, we have invested in good quality microphones for the students during the UATs and have consulted with software developers to use the latest voice-training feature. Early setups and test runs of the computers and VPs by the team are crucial before test runs with students.

This program of virtual learning aims to enhance learning and mimic reality to the technology’s best capacity. Although environmental and nonverbal cues were taken into consideration when programming the VPs’ settings and behaviors, these aspects of communication can be further refined during interactions between students and VPs by including action-related selections for users to select or active reflection elements that allow the users to reflect on time points in the conversation continuums, during which nonverbal communication and environmental factors could have made a difference. For instance, in the third scenario, although SBAR can be structural, much thought had to be put into the scenarios so that the conversations do not seem robotic or linear. The program must carry a certain caliber of conversational capacities and physical attractiveness to enchant users. Conversational elements are often unpredictable and arise from real user test runs of the program. Like previous scenarios, with a small team being familiar with the nursing context and conversational skills, variations may be limited. Future studies that are set in diverse and multicultural environments should consider including other languages or dialect inputs for their VP programs to simulate a more realistic hospital case setting for students.

### Expectations Management and Contingency Planning

A possible setback to the teamwork is the misunderstanding of expectations toward other expertise. The content creators were unsure of the processes needed to incorporate changes and the logic of the program operating system, whereas the IT team were unsure of the expectations of the outcome of the program. As the program is still in its preliminary stage, expectation management of the team is essential to keep the team grounded and prepared for potential hiccups. Establishing open and clear communication, setting realistic team and user expectations and deadlines, and having an administrative figure to keep track of deadlines and to keep everyone on track will help to facilitate an effective operation of the task and to ensure that all members are equally involved in the end goal. Meeting minutes should also be taken at the end of every meeting and sent to all members for reference. A consolidation of the deliverables should also be readily available and should be revisited before every subsequent meeting. It is important that before every UAT, clear instructions are given to the students that the purpose of using the VPs is to provide additional training to practice communication skills and not to replace actual or standardized patients. This may provide them with a sense of realism about the actual intent of the VCAAI program, which will thus avoid unnecessary comparisons and follow-on frustrations.

During the development stage, the team should prepare a blueprint by breaking the project into smaller parts, such as content development and IT development. This includes discussing possible exhaustive permutations during content development and having weekly meetings between the IT developers and the content developers to clarify any doubts and to document the process and progress of the project until its completion. There should be exigency plans throughout the development and execution phases of the VPs and UATs. Moreover, 2 of the most important lessons that were learned during the development phase of the VPs were to factor enough time for the content development and training of the VPs and to be prepared with alternatives. We also foresee challenges of recruiting and engaging students during the UATs as the test runs will take place over 4 different semesters of the nursing undergraduate program. Having strategic plans such as sending multiple reminders to the students before the UATs, providing them options of engaging in UATs even from home by downloading the provided links, and planning the UATs’ dates to match their academic timetables to avoid clashes with their other classes will be helpful.

### Conclusions

The adoption and creation of VP simulations to hone nursing students’ communication and interview skills with patients, health care providers, and colleagues will not only provide truly unique and authentic learning experiences for students but also potentially enhance their perceived self-efficacy and confidence in effective communication skills. However, given the infancy stage of this project, further refinements and constant enhancements are needed to train the VPs, such as by increasing possible permutations and improving speech-to-text translations to facilitate more realistic conversations. Future developers and users of the VCAAI can learn from the reflections shared by the authors to avoid similar pitfalls and enhance the user experience.

## Abbreviations

3D: three-dimensional
AI: artificial intelligence
COLDSPA: Characteristics of the pain, onset of the pain, location of the pain, duration of the pain, severity of the pain, precipitating factors that make the pain worse or make her feel better, and associated symptoms
IFE: Ideas or beliefs of cause of the situation, how the situation is affecting daily Function, and what Expectations one has from the interviewer over the situation
IT: information technology
MIRS: Master Interview Rating Scale
NURS: Naming emotion, express Understanding, showing Recognition, and offering Support
NUS: National University of Singapore
SBAR: situation, background, assessment, and recommendation
UAT: user acceptance test
VCAAI: virtual counseling application using artificial intelligence
VP: virtual patients
