# Metadata Correction: A Virtual Counseling Application Using Artificial Intelligence for Communication Skills Training in Nursing Education: Development Study

**DOI:** 10.2196/17064

**Published:** 2019-11-26

**Authors:** Shefaly Shorey, Emily Ang, John Yap, Esperanza Debby Ng, Siew Tiang Lau, Chee Kong Chui

**Affiliations:** 1 Alice Lee Centre for Nursing Studies National University of Singapore Singapore Singapore; 2 Information Techonology National University of Singapore Singapore Singapore; 3 Department of Mechanical Engineering National University of Singapore Singapore Singapore

In “A Virtual Counseling Application Using Artificial Intelligence for Communication Skills Training in Nursing Education: Development Study” by Shorey et al (J Med Internet Res 2019;21(10):e14658), the affiliation of authors Shefaly Shorey and Emily Ang has been corrected from “Department of Mechanical Engineering, National University of Singapore” to “Alice Lee Centre for Nursing Studies, National University of Singapore”.

The contact information for Shefaly Shorey has also changed from “Department of Mechanical Engineering, National University of Singapore, Singapore, Singapore” to “Alice Lee Centre for Nursing Studies, National University of Singapore, Clinical Research Centre 10 Medical Drive, Singapore, 117597, Singapore”.

The correction will appear in the online version of the paper on the JMIR website on November 26, 2019, together with the publication of this correction notice. Because this was made after submission to PubMed, PubMed Central, and other full-text repositories, the corrected article has also been resubmitted to those repositories.

